# Transcriptomic Responses to Coaggregation between Streptococcus gordonii and Streptococcus oralis

**DOI:** 10.1128/AEM.01558-21

**Published:** 2021-10-28

**Authors:** Siew Woh Choo, Waleed K. Mohammed, Naresh V. R. Mutha, Nadia Rostami, Halah Ahmed, Natalio Krasnogor, Geok Yuan Annie Tan, Nicholas S. Jakubovics

**Affiliations:** a Department of Biology, College of Science and Technology, Wenzhou-Kean University, Wenzhou, Zhejiang Province, People’s Republic of China; b Institute of Biological Sciences, Faculty of Science, University of Malaya, Kuala Lumpur, Malaysia; c School of Dental Sciences, Faculty of Medical Sciences, Newcastle University, Newcastle upon Tyne, United Kingdom; d Department of Basic Science, College of Dentistry, University of Anbar, Ramida, Anbar, Iraq; e Center for Excellence of Clinical Microbiome Research, All India Institute of Medical Sciences, Bhubaneswar, India; f Zhejiang Bioinformatics International Science and Technology Cooperation Centre Wenzhou-Kean University, Wenzhou, Zhejiang Province, People’s Republic of China; University of Manchester

**Keywords:** bioinformatics, coaggregation, oral streptococci, *Streptococcus gordonii*, *Streptococcus oralis*, transcriptome, biofilms

## Abstract

Cell-cell adhesion between oral bacteria plays a key role in the development of polymicrobial communities such as dental plaque. Oral streptococci such as Streptococcus gordonii and Streptococcus oralis are important early colonizers of dental plaque and bind to a wide range of different oral microorganisms, forming multispecies clumps or “coaggregates.” S. gordonii actively responds to coaggregation by regulating gene expression. To further understand these responses, we assessed gene regulation in S. gordonii and S. oralis following coaggregation in 25% human saliva. Coaggregates were formed by mixing, and after 30 min, RNA was extracted for dual transcriptome sequencing (RNA-Seq) analysis. In S. oralis, 18 genes (6 upregulated and 12 downregulated) were regulated by coaggregation. Significantly downregulated genes encoded functions such as amino acid and antibiotic biosynthesis, ribosome, and central carbon metabolism. In total, 28 genes were differentially regulated in Streptococcus gordonii (25 upregulated and 3 downregulated). Many genes associated with transporters and a two-component (NisK/SpaK) regulatory system were upregulated following coaggregation. Our comparative analyses of S. gordonii*-*S. oralis with different previously published S. gordonii pairings (S. gordonii*-*Fusobacterium nucleatum and S. gordonii*-*Veillonella parvula) suggest that the gene regulation is specific to each pairing, and responses do not appear to be conserved. This ability to distinguish between neighboring bacteria may be important for S. gordonii to adapt appropriately during the development of complex biofilms such as dental plaque.

**IMPORTANCE** Dental plaque is responsible for two of the most prevalent diseases in humans, dental caries and periodontitis. Controlling the formation of dental plaque and preventing the transition from oral health to disease requires a detailed understanding of microbial colonization and biofilm development. Streptococci are among the most common colonizers of dental plaque. This study identifies key genes that are regulated when oral streptococci bind to one another, as they do in the early stages of dental plaque formation. We show that specific genes are regulated in two different oral streptococci following the formation of mixed-species aggregates. The specific responses of S. gordonii to coaggregation with S. oralis are different from those to coaggregation with other oral bacteria. Targeting the key genes that are upregulated during interspecies interactions may be a powerful approach to control the development of biofilm and maintain oral health.

## INTRODUCTION

Oral streptococci, including Streptococcus gordonii and Streptococcus oralis, are among the most common bacteria in biofilms on the hard and soft tissues in the mouth (1). While S. gordonii predominantly colonizes tooth surfaces, S. oralis is frequently found both in dental plaque and in biofilms on soft tissues in the oral cavity (2, 3). Like many other oral streptococci, S. gordonii and S. oralis are able to adhere to cells of different species through specific adhesin-receptor interactions (4). Adhesion specificity is not fully conserved between different strains of a species due to differences in the key adhesins or receptors. For example, S. gordonii SK120 coaggregates with different strains of Actinomyces species compared with S. gordonii DL1 (Challis), M5, and SK184 (5). Genomic sequence analysis has revealed marked differences in the structure of a genetic locus encoding the coaggregation receptor polysaccharide (RPS) in S. gordonii SK120 compared with those of S. gordonii DL1, M5, and SK184, which likely underpins the differences in coaggregation specificity (6).

Many different coaggregation interactions can be detected between different strains of bacteria isolated from the mouth of an individual (7). It is thought that these interactions are critical for the colonization of surfaces in the mouth by microorganisms. For example, in a mouse model, the introduction of Candida albicans to the oral cavity in the absence of sucrose enhances mucosal biofilm formation by S. oralis (8, 9). S. gordonii expresses a range of cell surface adhesins that mediate adhesion with components of the acquired enamel pellicle, a layer of proteins and glycoproteins that coats the tooth surface (10). S. gordonii also adheres to a range of bacteria and is thought to be important for recruiting the periodontal keystone pathogen Porphyromonas gingivalis to dental plaque biofilms (11).

Coaggregation interactions will bring cells into close proximity with one another in oral microbial communities. It has been proposed that this enables cells to sense different species and to adapt in order to optimize their growth and survival within polymicrobial biofilms (12). Recently, a number of studies have investigated the impact of coaggregation or mixed-species biofilm formation on gene expression. S. gordonii has become a model organism for such studies due to its multifarious interactions with different partners. Thus, studies have explored interactions between S. gordonii and Aggregatibacter actinomycetemcomitans (13), C. albicans (14), P. gingivalis (15), or Actinomyces oris (16). However, each of these studies has used different models for bringing the cells together, and it is therefore difficult to identify genes that are regulated by cell-cell binding independently of the adhesion partner. In an attempt to standardize this approach, we have developed a simple method for studying coaggregation-mediated gene regulation by mixing suspensions of different bacteria in 25% human saliva to form coaggregates, incubating for 30 min, extracting RNA, and assessing gene expression by dual transcriptome sequencing (RNA-Seq). Using this approach, we have identified a number of genes that are regulated in S. gordonii in response to coaggregation with Fusobacterium nucleatum or Veillonella parvula (17, 18).

So far, studies on gene regulation responses to coaggregation have focused on intergeneric or interkingdom interactions. However, intrageneric coaggregation interactions have also been demonstrated. For example, a protein adhesin of S. gordonii DL1 recognizes RPS on the cell surface of S. oralis 34 that results in coaggregation (5, 19). Using antibodies against S. gordonii DL1 and the type of RPS expressed by S. oralis 34, interactions were also shown to occur between these bacteria in dental plaque developed *in situ* in the mouth of a volunteer (20). However, it is not yet clear whether the coaggregation between cells of the same genus results in cell-cell sensing and gene regulation in the partner organisms.

Here, we performed transcriptome profiling using a dual RNA-Seq approach to concurrently identify global changes in gene expression in S. gordonii DL1 and S. oralis 34 following coaggregation. This work builds on and improves our understanding of the interactions between S. gordonii and S. oralis and provides insights into their potential roles during the formation of mixed-species biofilms. We also compared these genes with the sets of genes that we identified in the interactions between S. gordonii and other bacterial species (e.g., F. nucleatum and V. parvula) in order to examine whether there are any mechanisms that are common among these S. gordonii*-*related bacterial pairings.

## RESULTS

### Generation of reference genome for S. oralis.

Due to the lack of a reference genome of *S oralis* 34, which was required for this transcriptomic study, we sequenced the genome of S. oralis 34 using Illumina HiSeq sequencing technology. The sequencing yielded 175,190 reads representing approximately 37-fold mean genome coverage. The assembly of these reads yielded six contigs with a GC content of 41.2% (see Table S1 in the supplemental material). The total assembly size was 1,904,876 bp with an *N*_50_ of 1,534,347 bp, suggesting that the assembly is suitable to be used as a reference genome for downstream transcriptomic analysis.

### Coaggregation of Streptococcus gordonii and Streptococcus oralis.

To assess the formation of coaggregates between S. gordonii and S. oralis, coaggregation was assessed semiquantitatively by vigorously mixing concentrated suspensions of cells in coaggregation buffer. Substantial aggregates were observed with a clear background and were scored “4+” on the visual coaggregation scale (21). Coaggregation was also monitored in freshly collected 25% human saliva; again, strong coaggregation was observed within seconds and was designated 4+ in reference to the standard visual scoring system.

**FIG 1:**
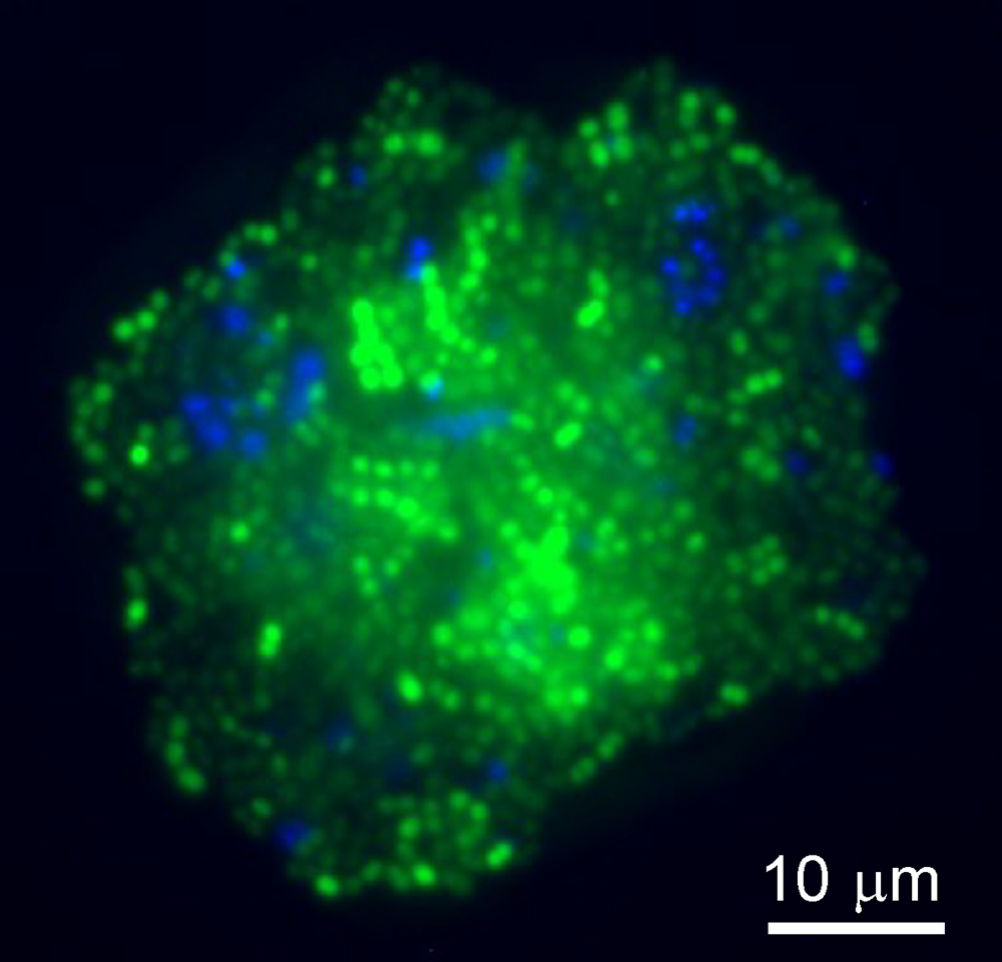
Visualization of coaggregation between Streptococcus gordonii and Streptococcus oralis. Example of a coaggregate formed between S. gordonii (Syto 9; green) and S. oralis (4′-6-diamidino-2-phenylindole [DAPI]; blue) in 25% human saliva. Cells were prestained before mixing and were visualized by fluorescence microscopy. The image shows a large aggregate.

To more closely assess the interactions between S. gordonii and S. oralis, prestained cells of each species were mixed in 25% human saliva to induce coaggregation and visualized by confocal laser scanning microscopy (Fig. 1). Large coaggregates were observed that contained at least 100 cells of each species. S. gordonii and S. oralis cells were interspersed throughout these structures, indicating that there was significant potential for cell-cell sensing and responses, as would occur in surface-associated biofilms. Therefore, to explore gene regulation in each species in response to coaggregation, monocultures and equivalent cultures containing coaggregated bacteria were set up in 25% human saliva, incubated for 30 min, harvested, and subjected to RNA extraction. The quality of each RNA preparation was assessed by NanoDrop spectroscopy, Bioanalyzer, and agarose gel electrophoresis, prior to sequencing on the Illumina HiSeq platform.

### Dual RNA-Seq data analysis.

Dual transcriptome sequencing was carried out with 5 or 6 independent biological repeats for monocultures or mixed cultures. A total of 16 sequencing libraries, comprising 5 mixed (S. gordonii-S. oralis), 6 monoculture S. gordonii, and 5 monoculture S. oralis biological replicates were sequenced, yielding approximately 339 million paired-end raw reads with read length of 100 bp (Table 1). After the removal of low-quality reads and adapter content by Trimmomatic v. 0.36, a total of approximately 314 million clean reads (clean ratio = 93%) were obtained.

**TABLE 1:** Mapping statistics of mixed and monoculture transcriptomes of S. gordonii and S. oralis in the coaggregation experiment

Sample name	No. of raw reads	No. of preprocessed reads	No. of mapped reads (%)
Monocultures			
S. gordonii			
Sg1	13,702,956	13,629,680	13,104,542
Sg2	15,266,848	15,185,702	14,640,981
Sg3	12,783,626	12,715,336	12,435,361
Sg4	34,884,736	32,450,626	31,924,924
Sg5	26,850,598	24,852,990	24,448,922
Sg6	30,372,844	28,134,060	21,986,544
All reads	133,861,608	127,187,392	118,194,982 (92.9%)
S. oralis			
So1	14,451,066	14,451,066	13,926,290
So2	15,621,300	15,619,918	14,879,371
So3	12,780,306	12,779,208	12,164,752
So4	32,061,362	29,339,116	28,867,869
So5	31,849,734	29,208,704	28,709,530
All reads	106,763,768	101,398,012	98,547,812 (97.1%)
Coaggregates			
SgSo1	13,414,958	4,269,588	1,095,399 (S. gordonii); 2,984,283 (S. oralis)
SgSo2	15,148,212	15,146,904	5,229,367 (S. gordonii); 9,382,155 (S. oralis)
SgSo3	12,743,546	12,742,346	5,885,024 (S. gordonii); 6,499,028 (S. oralis)
SgSo4	33,322,784	30,982,486	15,833,006 (S. gordonii); 15,370,738 (S. oralis)
SgSo5	24,359,624	22,479,16	15,833,006 (S. gordonii); 12,065,953 (S. oralis)
Mixed cultures	98,989,124	85,620,486	38,563,039 (45.04%; S. gordonii); 46,302,157 (54.08%; S. oralis)
All reads (monoculture and coaggregate)	339,614,500	314,205,890	301,607,990 (96%)

### Read mapping and transcript abundance estimation in mixed and monocultures.

For S. gordonii monoculture samples, approximately 92.9% of preprocessed reads were mapped to the reference genome of S. gordonii DL1 (NCBI accession number NC_009785.1), whereas for the S. oralis monoculture samples, 97.1% of the reads were successfully mapped to the assembled genome of S. oralis 34 (Table 1). The high mapping rate indicated that our sequencing data were high quality and suitable for downstream analyses. For mixed culture samples, we bioinformatically separated the read sequences of the two different transcriptomes by mapping the reads to the reference genomes of the two bacterial species. On average, 45% of the reads of the mixed cultures were mapped to the reference genome of S. gordonii (data set SgSo_Sg), and 54% of the reads were mapped to the reference genome of S. oralis (data set SgSo_So) (Table 1). Read counts of the mixed and monoculture samples were normalized using the trimmed mean of M values (TMM). The normalized distributions of data were comparable between the mixed and coaggregate and monoculture samples, and no apparent batch effects were observed (Fig. S1).

### Differential expression analysis in mixed and monocultures.

To investigate the impact of coaggregation on gene expression, differential gene expression analysis was performed using DESeq2 (22). Comparing the SgSo_So coaggregate and S. oralis monoculture identified 18 differentially expressed genes (6 upregulated and 12 downregulated) in S. oralis using a significance cutoff of a *P* value of <0.05 and a fold change of at least 2 (Table 2). After comparison between SgSo_Sg and *S. gordonii* monoculture, we identified 28 significant differentially expressed genes (25 upregulated and 3 downregulated genes) in S. gordonii (Table 3). The differentially expressed genes were visualized using volcano plots (Fig. S2).

**TABLE 2:** Full list of differentially expressed genes found in S. oralis

Identifier	Gene name	Protein name	Regulation	Fold change	*P*
13396_Soralis34_01263^*a*^	*rpsR*	30S ribosomal protein S18	Upregulated	4.11	6.12E−03
13396_Soralis34_01748^*a*^	*rpmGA*	50S ribosomal protein L33 1	Upregulated	2.67	1.90E−02
13396_Soralis34_00180^*a*^	*rpsS*	30S ribosomal protein S19	Upregulated	2.59	1.38E−03
13396_Soralis34_01130^*a*^	*rplL*	50S ribosomal protein L7/L12	Upregulated	2.42	4.55E−02
13396_Soralis34_00682			Upregulated	2.30	2.04E−02
13396_Soralis34_01310	*gpmA_3*	2,3-Bisphosphoglycerate-dependent phosphoglycerate mutase	Upregulated	2.25	2.78E−02
13396_Soralis34_01404^*b*^	*trpF*	*N*-(5′-phosphoribosyl)anthranilate isomerase	Downregulated	2.76	1.06E−02
13396_Soralis34_01406^*b*^	*trpD*	Anthranilate phosphoribosyltransferase	Downregulated	2.41	2.24E−04
13396_Soralis34_01408^*b*^	*trpE*	Anthranilate synthase component 1	Downregulated	2.39	3.52E−06
13396_Soralis34_01407	*folP*	Dihydropteroate synthase	Downregulated	2.38	1.92E−05
13396_Soralis34_01405^*b*^	*trpC*	Indole-3-glycerol phosphate synthase	Downregulated	2.27	9.91E−03
13396_Soralis34_00768	*opuBA_1*	Choline transport ATP-binding protein OpuBA	Downregulated	2.26	9.84E−03
13396_Soralis34_00766			Downregulated	2.24	2.39E−02
13396_Soralis34_00578			Downregulated	2.24	4.89E−02
13396_Soralis34_01403^*b*^	*trpB*	Tryptophan synthase beta chain	Downregulated	2.24	2.25E−02
13396_Soralis34_01314			Downregulated	2.15	3.81E−02
13396_Soralis34_01402^*b*^	*trpA*	Tryptophan synthase alpha chain	Downregulated	2.14	3.88E−02
13396_Soralis34_00767			Downregulated	2.09	4.86E−02

a Ribosomal proteins that were significantly upregulated in S. oralis in response to coaggregation with S. gordonii.

b Tryptophan metabolism genes that were significantly downregulated in S. oralis in response to coaggregation with S. gordonii.

**TABLE 3:** Full list of differentially expressed genes found in S. gordonii

Locus tag	Gene name	Protein name	Regulation	Fold change	*P*
SGO_RS09355^*a*^	SGO_1911	ABC-type transporter, ATPase component	Upregulated	18.15	1.08E−07
SGO_RS04515			Upregulated	14.94	1.68E−05
SGO_RS09350^*a*^	SGO_1910	Membrane protein, putative	Upregulated	12.15	1.32E−12
SGO_RS04530			Upregulated	10.52	3.86E−07
SGO_RS04520			Upregulated	5.50	1.01E−03
SGO_RS04535			Upregulated	4.10	3.50E−06
SGO_RS04525			Upregulated	3.98	2.98E−02
SGO_RS04510^*a*^	SGO_0920	Cobalt ABC transporter, ATP-binding protein	Upregulated	3.96	2.83E−23
SGO_RS04505^*a*^	SGO_0919	ABC transporter, ATP-binding protein	Upregulated	3.74	6.21E−17
SGO_RS05260	SGO_1071	Uncharacterized protein	Upregulated	3.07	1.87E−02
SGO_RS01830			Upregulated	3.06	8.47E−03
SGO_RS04500^*a*^		ABC-type transporter permease	Upregulated	3.02	1.25E−08
SGO_RS01720	SGO_0348	Reductase (EC 1.5.1.3)	Upregulated	2.90	2.19E−03
SGO_RS06370	SGO_1298	Uncharacterized protein	Upregulated	2.79	2.84E−02
SGO_RS08955	SGO_1825	Acetyltransferase, GNAT family	Upregulated	2.63	4.13E−02
SGO_RS03395	SGO_0689	Uncharacterized protein	Upregulated	2.62	3.96E−02
SGO_RS04495^*a*^	SGO_0917	Membrane protein, putative	Upregulated	2.61	6.98E−09
SGO_RS09340^*a*^	SGO_1908	DNA response regulator	Upregulated	2.53	1.04E−03
SGO_RS04115	SGO_0839	TfoX N-terminal domain superfamily	Upregulated	2.43	2.27E−02
SGO_RS01965	SGO_0394	Membrane protein, putative	Upregulated	2.37	3.54E−02
SGO_RS08490	SGO_1732	Histidine kinase (EC 2.7.3.–)	Upregulated	2.36	1.52E−07
SGO_RS04130			Upregulated	2.20	3.64E−02
SGO_RS02465	*dsg* (SGO_0498)	Putative permease	Upregulated	2.19	2.15E−03
SGO_RS01835			Upregulated	2.16	3.37E−02
SGO_RS04940	SGO_1008	Phosphohydrolase (MutT/nudix family protein) (EC 3.6.1.–)	Upregulated	2.02	1.47E−02
SGO_RS01520			Downregulated	4.85	2.69E−02
SGO_RS07630	SGO_1557	NrdH-redoxin	Downregulated	2.37	1.87E−03
SGO_RS01275			Downregulated	2.05	2.02E−04

a Transporter and two-component system genes that were upregulated in S. gordonii in response to coaggregation with S. oralis.

### Gene regulation in S. oralis in response to coaggregation with S. gordonii.

**FIG 2:**
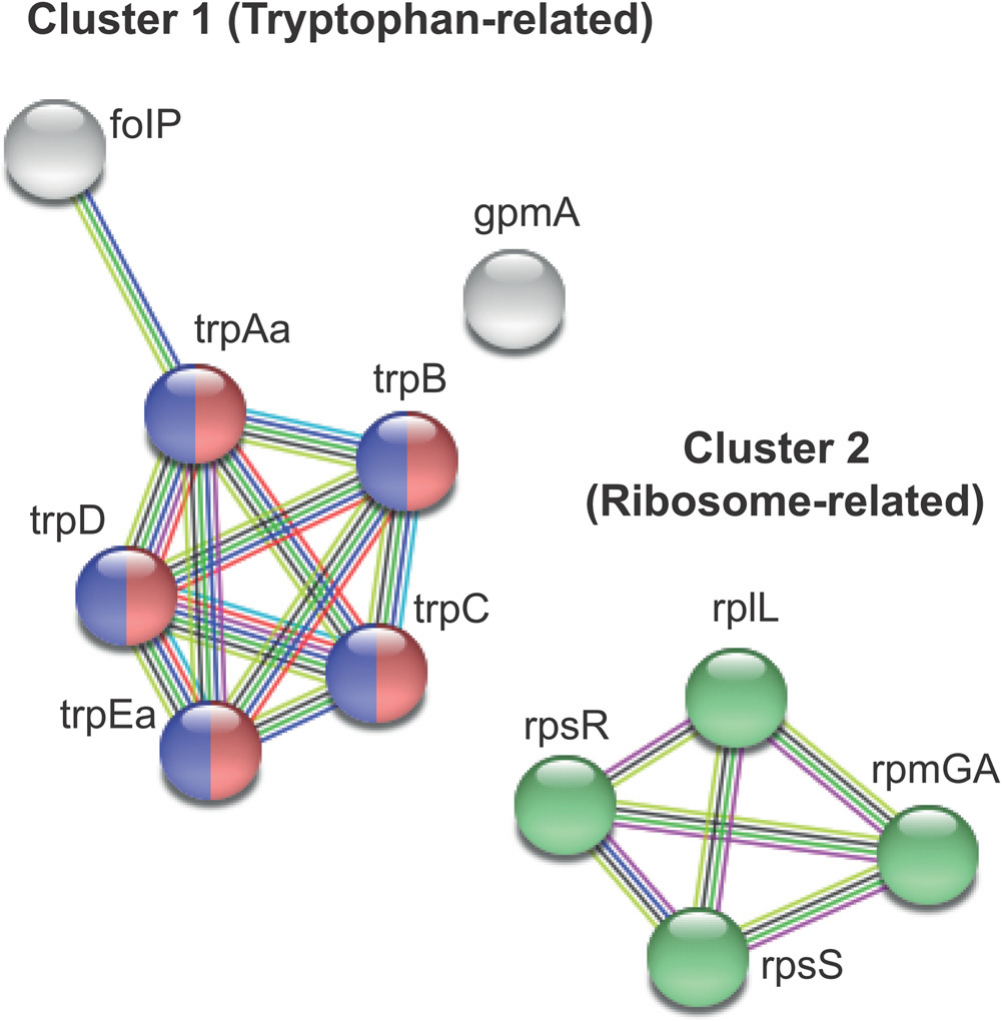
Network of genes regulated in S. oralis in coaggregation with S. gordonii visualized using the STRING database. Nodes were clustered by Markov cluster (MCL) clustering into three groups, represented by a single gene (*gpmA*, encoding 2,3-bisphosphoglycerate-dependent phosphoglycerate mutase) and two gene clusters indicated in circles of different colors. Interactions between nodes are depicted by colored solid lines. Different colors represent evidence from different sources, such as gene neighborhood (green), gene cooccurrence (dark blue), text mining (yellow), curated databases (cyan), experimentally determined (magenta), coexpression (black), protein homology (light blue), and gene fusions (red). Genes involved in phenylalanine, tyrosine, and tryptophan biosynthesis (red nodes), biosynthesis of amino acids (blue nodes), and ribosome (green nodes). Following coaggregation, all genes in cluster 1 were downregulated, whereas the genes in cluster 2 were upregulated in S. oralis.

To get better insights into the interactome of genes regulated in S. oralis, we performed a network analysis using STRING. The downregulated genes were mostly interacting and formed two prominent clusters. Cluster 1, the largest cluster, was comprised of downregulated genes involved in tryptophan biosynthesis (Fig. 2). Cluster 2 was comprised of the upregulated genes *rpsS* (S19 protein), *rpsR* (S18 protein), *rpmGA* (L33 protein), and *rpsL* (L7/L12 protein), encoding ribosomal proteins that were upregulated from 2.42- to 4.11-fold (Table 2). In each of these clusters, gene interactions were based on multiple lines of evidence indicating that they are likely to be functionally related. The STRING functional enrichment analysis revealed three major functions: phenylalanine, tyrosine, and tryptophan biosynthesis (false-discovery rate [FDR] = 1.08E−06), biosynthesis of amino acids (FDR = 0.00024), and ribosome (FDR = 0.00076) (Fig. 2). Cluster 1 genes involved in phenylalanine, tyrosine, and tryptophan biosynthesis were downregulated between 2.14 and 2.76-fold in S. oralis following coaggregation (Table 2). The genes in this pathway are all involved in the tryptophan biosynthesis pathway.

### Gene regulation in S. gordonii in response to coaggregation with S. oralis.

**FIG 3:**
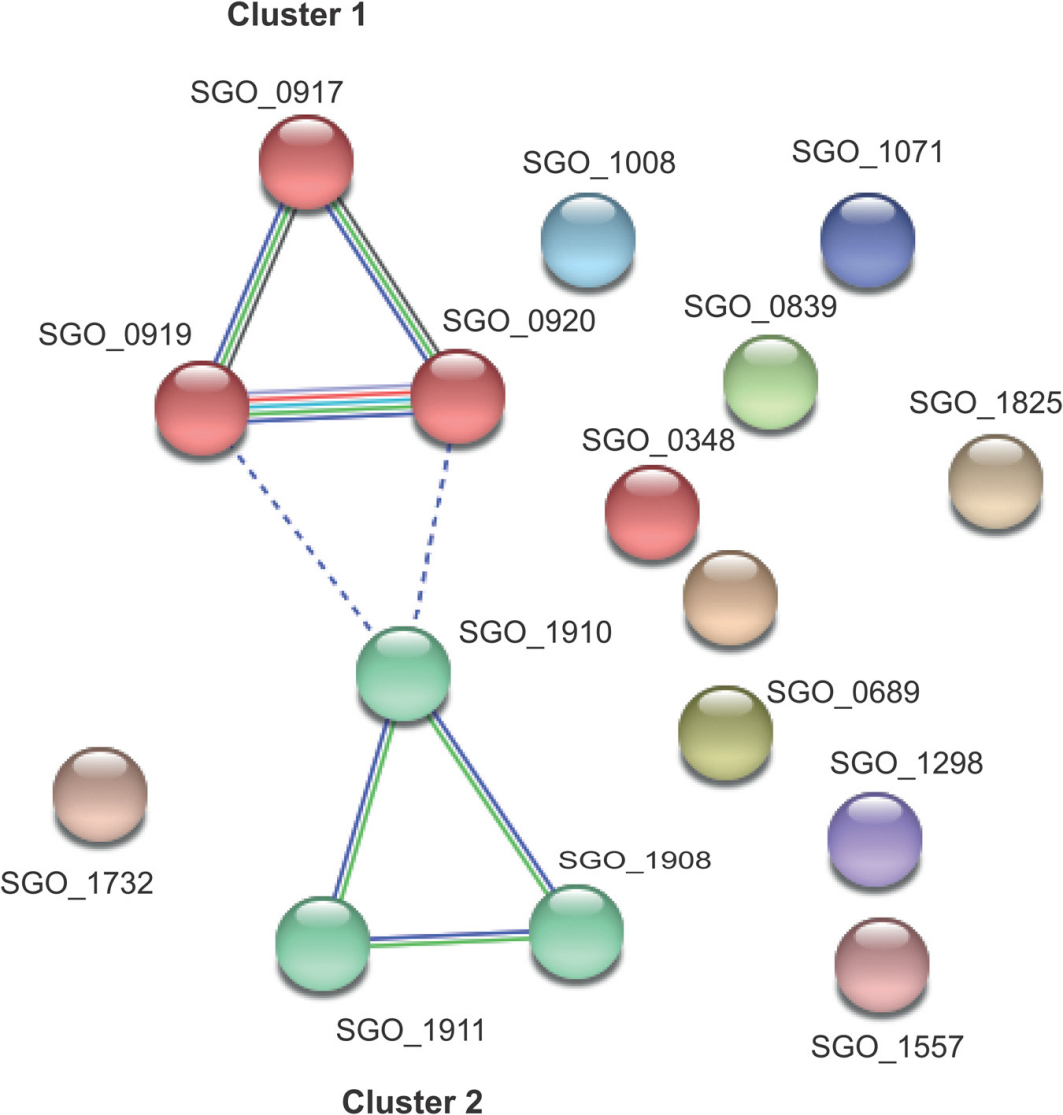
Network of genes regulated in S. gordonii in coaggregation with S. oralis. The largest connected group of genes encodes components of transporters (Table 2). Nodes were clustered by MCL clustering into two groups (cluster 1 and cluster 2). Interactions between nodes are depicted by colored lines. Different colors represent evidence from different sources such as gene neighborhood (green), gene cooccurrence (dark blue), text mining (yellow), curated databases (cyan), experimentally determined (magenta), coexpression (black), protein homology (light blue), and gene fusions (red). All genes in cluster 1 were upregulated in S. gordonii following coaggregation. Solid lines indicate interactions between genes in the same gene cluster, whereas dotted lines indicate interactions between genes in different gene clusters.

Genes regulated in S. gordonii were dominated by transporter genes and particularly ATP-binding cassette (ABC)-type transporters (Table 3). Two clusters encoding transporters, which included a two-component (NisK/SpaK) regulatory system, were upregulated in response to coaggregation (Fig. 3). Two-component systems consist of a transmembrane sensor and response regulator that induce or repress transcription of target genes in response to an external stimulus (23, 24). A tblastn homology analysis of our two-component system showed 33% to 35% similarity with Lactococcus lactis and Streptococcus suis NisK/NisR systems, which are involved in sensing lantibiotics. Mature lantibiotics in streptococci can be sensed by two-component systems, leading to an autoinduction process that results in the production and activation of lantibiotics in neighboring cells (25).

### Comparative analysis between different S. gordonii pairings.

**FIG 4:**
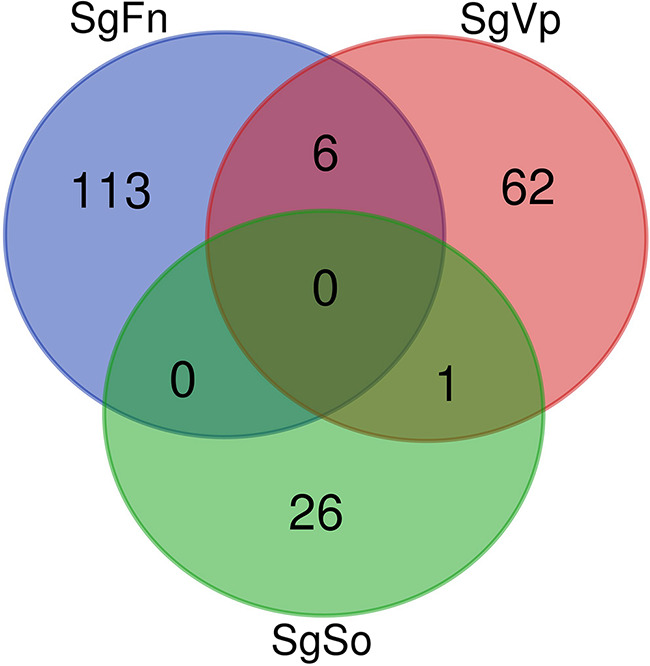
Venn diagram showing overlaps between three S. gordonii pairings. In total, six genes were common between S. gordonii-Fusobacterium nucleatum (SgFn) and S. gordonii-Veillonella parvula (SgVp), whereas only one gene was found in common between SgVp and S. gordonii-S. oralis (SgSo), and there were no genes in common between the SgSo versus SgFn pairings (see Table S2 in the supplemental material). Among the six genes that were regulated in SgFn and SgVp pairings, four genes were regulated in the same direction in two different bacterial pairings. These genes encode the tagatose-6-phosphate kinase (fold change = −3.2 in SgFn and −2.72 in SgVp), truncated hypothetical protein (fold change = 2.5 in SgFn and 2.62 in SgVp), short-chain dehydrogenase (fold change = −2.32 in SgFn and −2.75 in SgVp), and thiamine biosynthesis protein (fold change = −2.46 in SgFn and −2.04 in SgVp). Interestingly, two genes were regulated in the reverse direction in two different bacterial pairings. These genes encode the recombination regulator SGO_RS03085 (fold change = 2.84 in SgFn and −2.88 in SgVp) and Fur family transcriptional regulator (fold change = 2.42 in SgFn and −2.68 in SgVp). The one gene (pf08796 family protein) that was common between the SgSo and SgVp pairings was regulated in the same direction in both pairings.

We assessed the impact of coaggregation between S. gordonii and two key initial colonizers of dental plaque, Fusobacterium nucleatum and Veillonella parvula, on gene expression in each partner using the same approach described in this study (17, 18). We hypothesized that there are common mechanisms or pathways that are regulated in S. gordonii in response to coaggregation, independently of partner species. To examine this, we compared the differentially expressed genes of S. gordonii in each pairing (S. gordonii*-*S. oralis [SgSo], S. gordonii*-*F. nucleatum [SgFn], and S. gordonii*-*V. parvula [SgVp]) (Fig. 4). None of the genes were regulated commonly by coaggregation in all three bacterial pairings (Table S2).

It was noteworthy that the pairing with the evolutionarily most distant species, F. nucleatum, led to the highest number of S. gordonii genes regulated (119 genes). The pairing with V. parvula, another member of the phylum Firmicutes, led to regulation of 69 genes, whereas only 27 genes were regulated in S. gordonii following interactions with S. oralis. F. nucleatum cells are long and may bind multiple S. gordonii cells, further enhancing the potential to trigger gene regulation.

## DISCUSSION

Coaggregation has been suggested to play a key role in promoting interactions between different bacteria that lead to profound phenotypic changes in the partner cells that enable them to proliferate in biofilm formation. Previous studies have shown that cell-cell interactions during coaggregation or biofilm formation lead to changes in gene expression in the partner organisms that may be important for adaptation to a community lifestyle (14, 15, 26, 27). Here, we studied the interaction between S. gordonii and S. oralis. Both were shown to form 3-dimensional coaggregate structures with cells of different species that were relatively evenly spread throughout. This is similar to the arrangements of cells that we previously observed in S. gordonii-F. nucleatum and S. gordonii-*V. parvula* coaggregates (17, 18). The close proximity of different cell types in these structures facilitates the exchange of signals or cues that modulate cell-cell sensing and gene regulation.

Interestingly, our analysis showed the downregulation of a cluster of tryptophan biosynthesis pathway-related genes in S. oralis. This cluster of genes was recently identified in S. oralis subsp. *tigurinus* (formerly Streptococcus tigurinus [27]) and S. gordonii DL1 and was suggested to represent a novel pathway for production of indole. In some Gram-negative bacteria, tryptophan and indole play important roles in cell-cell communication and biofilm formation (28). For example, the production of indole by Escherichia coli interferes with cell-cell communication pathways of Pseudomonas aeruginosa and promotes the growth of E. coli in mixed cultures (29). On the other hand, tryptophan inhibits biofilm formation by both E. coli and P. aeruginosa (30, 31). Recently, indole has been shown to enhance biofilm formation by the cariogenic oral bacterium Streptococcus mutans (32). It is possible that the exchange of tryptophan and/or indole between S. gordonii and S. oralis may modulate cell-cell sensing and biofilm formation.

The downregulation of ribosomal protein expression has previously been shown to be associated with growth rate (33). A similar effect of downregulation on S. oralis ribosomal proteins by Anaeroglobus geminatus has been demonstrated in proteomic analysis of polymicrobial biofilm model (34). It is possible that competition from S. gordonii led to a decrease in the rate of S. oralis growth in coaggregates, although the short timescale of the experiments here did not allow the measurement of growth rate. It is noteworthy that a decreased protein synthesis rate has been shown to be linked to expression of tryptophan biosynthesis genes. Thus, it was shown that *trp* genes were downregulated when protein synthesis was reduced in Escherichia coli (35). Therefore, the coaggregation-mediated downregulation of the *trp* operon in S. oralis may be linked to a more general decrease in growth rate.

It can be hypothesized that proximity of S. gordonii and S. oralis in coaggregates may enhance the interbacterial competition between them, resulting in upregulation of sensing systems that detect competitive molecules such as lantibiotics. However, at present there is no experimental evidence regarding the role of this two-component system in S. gordonii, and further work is needed to confirm a function in sensing antimicrobial peptides.

Our data suggest that the gene regulation is very specific to each pairing and that responses do not appear to be conserved. This indicates that the process of aggregation and the resultant increase in cell density is not the main driver behind gene regulation, even though autoaggregation has been shown to lead to changes in gene expression in other bacteria, such as F. nucleatum (36). This ability to distinguish between neighboring bacteria may be important for S. gordonii to adapt appropriately during the development of complex biofilms such as dental plaque. It is interesting that stronger gene regulation was observed in the pairing with the most distantly related microorganism (F. nucleatum), and the lowest regulation was observed with the intrageneric interaction (S. oralis). It is important to note that the absolute number of genes regulated is highly dependent on the thresholds applied and can be influenced by batch effects. A more rigorous comparison of gene regulation during interactions with a wider range of different oral microorganisms in experiments performed alongside one another is required to show whether the extent of gene regulation following cell-cell interactions is associated with evolutionary distance between the partner strains.

This study and our previous two S. gordonii pairing studies described a range of genes and pathways in S. gordonii-F. nucleatum, S. gordonii-V. parvula, and S. gordonii-S. oralis in response to coaggregation with each other (17, 18). Coaggregation was successfully employed as a model to interpret transcriptional changes involved in biofilm formation. Oral streptococci may have hundreds of different coaggregation partners in the oral cavity (37, 38). Our work indicates that the transcriptional responses of streptococci such as S. gordonii will be highly dependent upon their cell-cell interactions as oral biofilms develop. Consequently, it may be difficult to identify genes that are critical for biofilm development under all conditions and that may be targeted for biofilm control approaches. Nevertheless, more detailed analyses of transcriptomic and metatranscriptomic changes during the formation of dental plaque will continue to provide insights into how different species of oral bacteria adapt to the formation of polymicrobial communities.

## MATERIALS AND METHODS

### Routine culture of bacteria.

S. gordonii DL1 (Challis; ATCC 35105) and S. oralis 34 (formerly S. sanguis 34) (39) were routinely cultured statically at 37°C in THYE medium consisting of Todd Hewitt Broth (30 g · liter^−1^; Difco, Becton, Dickinson and Company, Oxford, UK) and yeast extract (5 g · liter^−1^; Melford Laboratories, Ipswich, UK) or on solidified THYE medium containing Bacto agar (15 g · liter^−1^; Difco, Becton, Dickinson). Alternatively, bacteria were cultured in BHYG medium containing (per liter) 37 g brain heart infusion (Becton, Dickinson), 5 g yeast extract, 2.5 g sodium glutamate (Sigma-Aldrich, Dorset, UK). All media were sterilized by autoclaving at 121°C for 15 min before use. For long-term storage, stocks of bacteria were maintained at −80°C in THYE medium supplemented with 50% glycerol. The purity of cultures was checked frequently by phase-contrast microscopy and by plating aliquots on agar plates.

### DNA extraction and whole-genome sequencing.

Genomic DNA was purified from a 20-ml culture of S. oralis 34 using the MasterPure complete DNA and RNA purification kit (Epicentre Biotechnologies, Madison, WI) as instructed by the manufacturer. The extracted DNA was checked by agarose gel electrophoresis and NanoDrop spectrophotometry prior to being sent to the sequencing service group, MicrobesNG, at the University of Birmingham for sequencing. The sequencing was done using the Illumina HiSeq 2500 platform with a paired-end strategy with 100-bp reads. The *de novo* genome assembly was done using SPAdes v. Dec-2017 (40).

### Saliva preparation.

Ethical approval for the collection of saliva from healthy volunteers was obtained from the Newcastle University Research Ethics Committee (reference 14898/2018). All saliva donors were given a participant information sheet and gave written informed consent to participate in the study. Saliva was collected and pooled from five healthy individuals who had not eaten for at least 2 h prior to collection. Saliva was stimulated by chewing on Parafilm and was placed on ice immediately after collection. The reducing agent dithiothreitol (DTT) was added to a final concentration of 2.5 mM, and saliva samples were gently stirred on ice for 10 min. Aggregated particles were removed by centrifugation at 15,000 × *g* and 4°C for 30 min. Three volumes of H_2_O were added to 1 volume of saliva and sterilized by filtration through a 0.22-μm-pore membrane. Aliquots were stored at −20°C. The 25% saliva was thawed at 37°C immediately before use and any precipitate that had formed was removed by centrifugation at 1,400 × *g* and 20°C for 10 min.

### Coaggregation assays.

S. gordonii DL1 and S. oralis 34 were cultured at 37°C in THYE medium for 18 h, harvested by centrifugation at 3,800 × *g* for 10 min, and washed three times with one volume of phosphate-buffered saline (PBS; pH 7.3). Cells were resuspended in one volume of PBS and adjusted to an optical density at 600 nm (OD_600_) of 1.0 to give a final concentration of approximately 1 × 10^9^ CFU/ml. To visualize S. gordonii cells, Syto 9 (Life Technologies Ltd., Paisley, UK) was added to cells to achieve 7.5 μM. S. oralis 34 was stained by addition of 4′-6-diamidino-2-phenylindole (DAPI) (2.5 μg/ml final concentration; Thermo Scientific) in 1 ml of PBS solution containing bacterial cells. Cells were incubated at 37°C in the dark for 10 min. Fluorescently stained bacteria were washed twice with PBS and resuspended in 1 ml of 25% cell-free saliva. To induce coaggregation in dual-species cultures, 500 μl of each species were mixed by vortex for 10 s in glass test tubes and gently rocked by hand until coaggregation was visible. Samples were visualized using a 60× lens objective on an Olympus BX61 microscope (Olympus Corporation, Tokyo, Japan), equipped with a dichroic mirror to split the excitation and emission wavelengths. Images were captured using an Olympus XM10 monochrome camera.

To assess gene regulation responses to coaggregation, S. gordonii and S. oralis were cultured for 18 h at 37°C in BHYG medium, subcultured into fresh medium, and grown at 37°C to the mid-exponential phase (OD_600_ = 0.4 to 0.6). Cells were harvested at 3,800 × *g* and 20°C in a swing-out rotor for 10 min and adjusted to an OD_600_ of 1.0 ± 0.2. A 5-ml aliquot of each culture was harvested at 3,800 × *g* and 20°C for 5 min and resuspended in 0.5 ml of 25% saliva. Samples were divided into two equal portions. One was used for monoculture controls, while the other samples of each species were mixed together. Samples were mixed vigorously using a vortex mixer for 10 s. All samples were made up to 5 ml by the addition of 25% saliva and were incubated at 37°C for 30 min. RNAlater (5 ml; Invitrogen) was added, and the tubes were vortex mixed for 5 s and incubated at 20°C for 5 min. Cells were harvested at 3,000 × *g* for 15 min at 20°C, and the pellets were frozen at −80°C for subsequent RNA extraction.

### RNA-Seq data sets.

Six biological replicates of S. gordonii monoculture, five replicates of S. oralis monoculture, and five replicates for the S. gordonii-S. oralis mixed culture were used. In total, 16 samples were used in this study.

### RNA extraction.

To disrupt cells for RNA extraction, samples were thawed at 20°C and resuspended in 100 μl spheroplasting buffer containing 0.1 mg/ml spectinomycin (41). Mutanolysin was added to 500 U/ml, and cells were incubated at 37°C for 5 min. Total RNA was extracted using the Ambion RiboPure bacteria RNA purification kit (Life Technologies) according to the manufacturer’s instructions. RNA concentrations were determined using a NanoDrop ND-1000 spectrophotometer (Thermo Scientific). To ensure that RNA had not degraded during extraction, an aliquot of each sample was analyzed by gel electrophoresis.

### Library preparation and whole-transcriptome sequencing.

Library preparation and sequencing were performed by the established and internationally recognized sequencing provider BGI Tech Solutions (Hong Kong). Following an initial rRNA depletion, first-strand cDNA synthesis was performed using random hexamer primers. The second-strand cDNA was synthesized using buffer, deoxynucleoside triphosphates (dNTPs), RNase H, and DNA polymerase I, respectively, after removing dNTPs. Short fragments were purified with the QIAquick PCR extraction kit and resuspended in elution buffer for end repair and addition of poly(A) tails. The short fragments were ligated to sequencing adapters. Uracil *N*-glycosylase enzyme was used to degrade the second-strand cDNA, and products were purified by MinElute PCR purification kit before PCR amplification. All libraries were sequenced using the Illumina HiSeq 2500 platform with a paired-end sequencing strategy.

### Read mapping and preprocessing.

All raw reads generated from Illumina sequencing platform were preprocessed before mapping to reference genomes. Illumina adapters and low-quality reads (*Q* < 20) were removed with Trimmomatic v. 0.36. FastQC was used to verify removal of low-quality reads and adapters. Reads from S. gordonii monoculture were mapped to the NCBI reference genome (accession number NC_009785.1), whereas the reads from S. oralis were mapped to the assembled genome of S. oralis 34 that we sequenced in this study, using TopHat v1.0.14 with default parameters. Five replicates of mixed samples were mapped separately in two rounds to the reference genomes of S. gordonii and S. oralis and designated “SgSo_Sg” (reads of coaggregate culture mapped to S. gordonii reference genome from NCBI) and “SgSo_So” (reads of coaggregate culture mapped to S. oralis). After read mapping, SAMtools (42) was employed to calculate mapping statistics.

### Gene expression quantification, normalization, and differential expression analysis.

All mapped reads were used for quantifying gene expression using HTseq-count. HTseq (43) required a gene feature format (GFF) annotation file (mode = union, -t = gene, -i = locus_tag), and the standard gene annotations provided with reference genomes were used. Box plots were generated using in-house scripts to evaluate whether the normalization works well for all samples before downstream analyses. Comparisons were made between monoculture (S. gordonii or S. oralis) and coaggregate samples (SgSo_Sg and SgSo_So). Differential expression analyses between monoculture (S. gordonii or S. oralis) and coaggregate samples (SgSo_Sg and SgSo_So) were performed using the Bioconductor package *DESeq* v. 3.854 in the R statistical software program. DESeq-normalized gene count data were based on “size factors” to account for RNA-Seq library size differences, and dispersion estimates were calculated. Pairwise comparisons of expression were made between the monoculture and mixed-sample group for every replicate based on a negative binomial model. Fold changes were obtained along with their associated *P* values. A gene was defined as significantly expressed if it had a *P* value of <0.05 and a fold change of at least 2.

### STRING interaction network analysis.

The STRING v. 11.0 database was used to predict if there were any functional associations of differentially regulated significant genes (44). The search tool for retrieval of interacting genes/proteins (STRING) was used to identify known and predicted interactions based on evidence from different sources such as experiments, databases, neighborhood, text mining, cooccurrence, coexpression, gene fusion, and databases) using default settings. Nodes represent differentially expressed genes, and edges indicate the level of confidence in the association, with thicker lines indicating greater confidence. The network was clustered using the Markov cluster (MCL) clustering method with a specified “MCL inflation parameter” of 3. Kyoto Encyclopedia of Genes and Genomes (KEGG) pathway enrichment analysis was performed using STRING.

### Comparative analysis of S. gordonii in response to coaggregation with F. nucleatum, V. parvula, or S. oralis.

Using a Venn diagram, the S. gordonii genes that were common to three pairs of comparisons and the genes that were shared between any two pairs were identified. The genes commonly expressed from the three pairings were further investigated with STRING database analysis to explain possible common genes and pathways.

### Data availability.

Raw sequence reads were deposited in the NCBI Sequence Read Archive (SRA) database under accession numbers SRR12650300, SRR12650301, SRR12650302, SRR12650303, SRR12650304, SRR12650305, SRR12650306, SRR12650307, SRR12650308, SRR12650309, SRR12650310, SRR12650311, SRR12650312, SRR12650313, SRR12650314, and SRR12650315. The genome sequence of S. oralis 34 can be accessed in the GenBank database under accession number JAHKGX000000000.
