# Supplementary material for: Transcriptomic Responses to Coaggregation between Streptococcus gordonii and Streptococcus oralis
Source: Appl Environ Microbiol. 2021 Oct 28;87(22):e01558-21. doi: 10.1128/AEM.01558-21 (PMC8552878; doi:10.1128/AEM.01558-21)
Supplement: Supplemental file 1 — Tables S1 and S2, Fig. S1 and S2. Download AEM.01558-21-s0001.pdf, PDF file, 0.1 MB [file aem.01558-21-s0001.pdf]

Transcriptomic Responses to Coaggregation between *Streptococcus gordonii* and *Streptococcus oralis*

Siew Woh Choo, Waleed K. Mohammed, Naresh V. R. Mutha, Nadia Rostami, Halah Ahmed, Natalio Krasnogor, Geok Yuan Annie Tan, Nicholas S. Jakubovics

Supplementary Information

Supplementary Table 1. Genome statistics of *S. oralis*34. All statistics are based on contigs of size >= 500 bp, unless otherwise noted (e.g., "# contigs (>= 0 bp)" and "Total length (>= 0 bp)" include all contigs.)

**Statistics without reference**	
# contigs	6
# contigs (>= 0 bp)	25
# contigs (>= 1000 bp)	5
Largest contig	1534347
Total length	1904876
Total length (>= 0 bp)	1912045
Total length (>= 1000 bp)	1904349
N50	1534347
N75	1534347
L50	1
L75	1
GC (%)	41.2

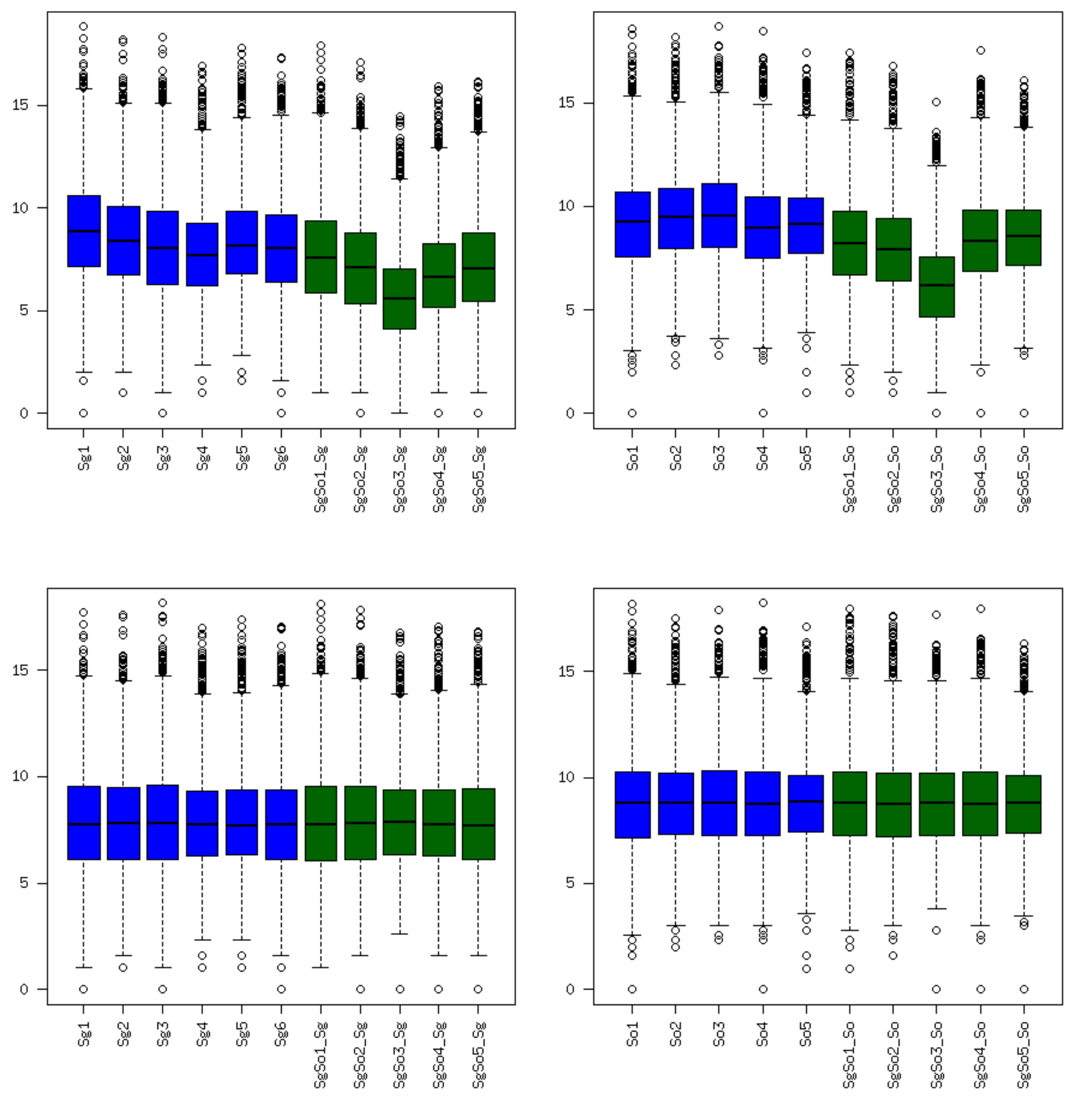

Supplementary Figure 1: Distribution of normalized read counts in SgSo experiments.

Counts were normalized by the Trimmed Mean of M-values approach. Median counts from each sample are shown as thick black lines surrounded by a box representing the interquartile range, with whiskers that extend to two standard deviations from the median. Outlying observations are shown as open circles. Data from biological replicates for each sample are shown.

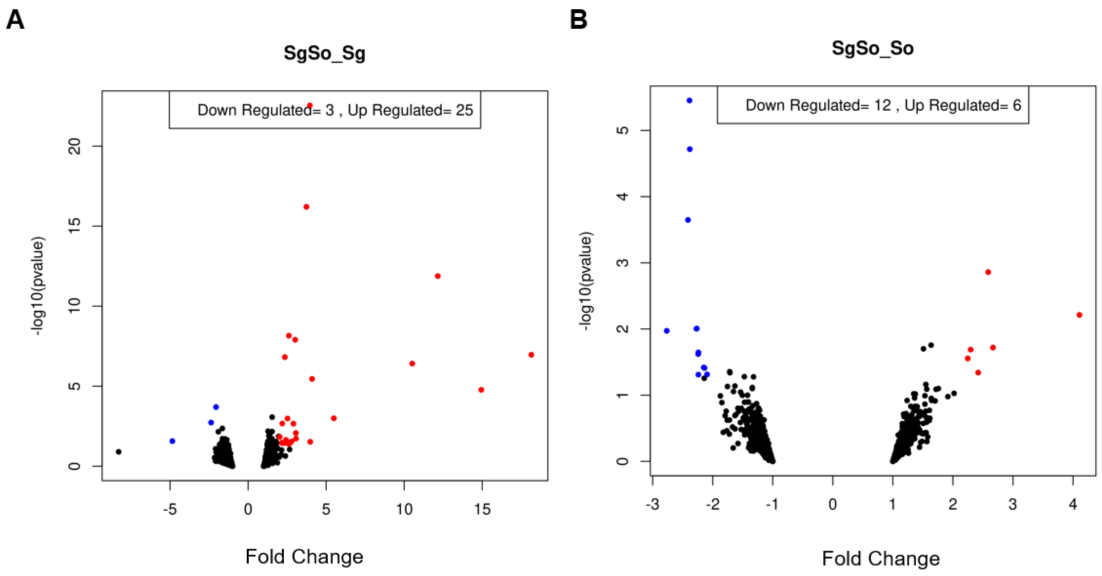

Supplementary Figure 2: Volcano plots representing differentially expressed genes in coaggregates vs monocultures for (A) *S. gordonii* and (B) *S. oralis.* The y-axis corresponds to the mean expression value of $\log_{10}$ (P value), and the x-axis displays the $\log_2$ (fold change) value. The green and red dots correspond to significantly down and up-regulated genes, respectively. The black dots represent genes that are not significantly expressed.

Supplementary Table 2. List of common *S. gordonii* genes between the three pairings of SgFn, SgVp and SgSo.

Comparisons	No. of Genes	Locus Tag	Gene Description	Fold Change SgFn/SgVp/SgSo
SgFn vs SgVp vs SgSo	0	-	-	-
SgFn vs SgVp	6	SGO_RS10595	truncated hypothetical protein	2.5/2.62
		SGO_RS03085	recombination regulator	2.84/-2.88
		SGO_RS04880	short-chain dehydrogenase	-2.32/-2.75
		SGO_RS05750	thiamine biosynthesis protein	-2.46/-2.04
		SGO_RS03460	Fur family transcriptional regulator	2.42/-2.68
		SGO_RS07435	tagatose-6-phosphate kinase	-3.22/-2.72
SgFn vs SgSo	0		-	-
SgSo vs SgVp	1	SGO_RS03395	pf08796 family protein	2.89/3.031
